# Understanding delays in chronic limb‐threatening ischaemia care: Application of the theoretical domains framework to identify factors affecting primary care clinicians' referral behaviours

**DOI:** 10.1002/jfa2.12015

**Published:** 2024-05-04

**Authors:** Eleanor Atkins, Panagiota Birmpili, Ian Kellar, Amundeep S. Johal, Qiuju Li, Sam Waton, Jonathan R. Boyle, Arun D. Pherwani, Ian Chetter, David A. Cromwell

**Affiliations:** ^1^ Clinical Effectiveness Unit Royal College of Surgeons of England London UK; ^2^ Hull York Medical School Hull UK; ^3^ University of Sheffield Sheffield UK; ^4^ Department of Health Services Research and Policy London School of Hygiene and Tropical Medicine London UK; ^5^ Department of Vascular Surgery Cambridge University Hospitals NHS Trust & Department of Surgery University of Cambridge Cambridge UK; ^6^ Staffordshire & South Cheshire Vascular Network Royal Stoke University Hospital Stoke‐on‐Trent UK

**Keywords:** chronic limb‐threatening ischaemia, pathways, primary care, referral, vascular surgery

## Abstract

**Introduction:**

Patients in the community with suspected Chronic limb‐threatening ischaemia (CLTI) should be urgently referred to vascular services for investigation and management. The Theoretical Domains Framework (TDF) allows identification of influences on health professional behaviour in order to inform future interventions. Here, the TDF is used to explore primary care clinicians' behaviours with regards to recognition and referral of CLTI.

**Methods:**

Semi‐structured interviews were conducted with 20 podiatrists, nurses and general practitioners in primary care. Directed content analysis was performed according to the framework method. Utterances were coded to TDF domains, and belief statements were defined by grouping similar utterances. Relevance of domains was confirmed according to belief frequency, presence of conflicting beliefs and the content of the beliefs indicating relevance.

**Results:**

Nine TDF domains were identified as relevant to primary care clinicians: Knowledge, Environmental context and resources, Memory, Decision and attention processes, Beliefs about capabilities, Skills, Emotions, Reinforcement and Behavioural regulation. Relationships across domains were identified, including how primary care clinician confidence and working in a highly pressurized environment can affect behaviour.

**Conclusion:**

We have identified key barriers and enablers to timely recognition and referral behaviour. These beliefs identify targets for theory‐driven behaviour change interventions to reduce delays in CLTI pathways.

**Abbreviations**
A&E: accident and emergency
ABPI: ankle–brachial pressure index
CLTI: chronic limb‐threatening ischaemia
COPD: chronic obstructive pulmonary disease
COREQ: consolidated criteria for reporting qualitative research
ESVS: European Society of Vascular Surgery
GP: general practitioner
LMC: local medical committee
PAD: peripheral arterial disease
PIS: participant information sheet
TDF: theoretical domains framework
VSGBI: Vascular Society of Great Britain and Ireland

## 1 INTRODUCTION

Chronic limb‐threatening ischaemia (CLTI) is the end stage of peripheral arterial disease (PAD) and is associated with significant morbidity and mortality [1]. Symptoms caused by the lack of blood supply to the lower limb include pain at rest, pain at night time preventing sleep and/or non‐healing ulceration or gangrene. Where possible, vascular surgeons aim to revascularize the affected limb, and these procedures are associated with improved mortality and limb salvage [2].

Early referral of suspected CLTI is important, as delays in revascularization are associated with increased mortality and limb loss [3]. The Vascular Society of Great Britain and Ireland (VSGBI) have released guidance stating patients with suspected CLTI should be referred to vascular surgery services on the same day they are seen and recognized as such by a clinician in the community [4]. However, delays exist at every point along the patient pathway from symptoms developing to revascularization, and there are missed opportunities to identify CLTI in primary care [5, 6].

Previous studies have suggested both patient factors, such as age, deprivation and delay in presentation, and primary care clinician factors, such as lack of awareness of guidelines and reliance on ankle‐brachial pressure index (ABPI), can affect timely referral [6, 7, 8]. Clinician education has previously been called for in order to improve referrals for PAD [9, 10, 11]. None of these studies, however, used theory or a theoretical framework to reach their conclusions.

The Theoretical Domains Framework (TDF) was developed in 2005 in order to integrate and simplify behaviour change theories, making theory more accessible to other disciplines [12]. It allows identification of influences on health professional behaviour related to implementation [13]. The TDF enables understanding of implementation problems and potential solutions [14]. It has been used in the past to understand blood transfusion behaviour in clinicians [15], to identify barriers and enablers for GP referrals for pulmonary rehabilitation [16, 17] and to understand other complex, multilevel behaviours such as prescribing [18]. If a theoretical approach is not taken to understand implementation difficulties, there will be limited opportunities to understand behaviour change and optimize resulting interventions [19].

CLTI should be treated urgently to improve chances of limb salvage and survival. An understanding of the factors influencing its recognition and referral from primary care is important in order to inform future strategies to reduce delay in the referral process. This qualitative study uses a theoretical approach to establish an evidence base in order to increase understanding of the primary care clinician‐reported factors affecting timely referral for suspected CLTI, with a view to developing future interventions. Difficulty changing behaviour is often the reason for failure of recommendations in guidelines to be translated into practice in healthcare [20], but using theory in the design of complex interventions increases the likelihood that they are successful in changing future behaviour [21, 22].

## 2 METHODS

### 2.1 Design

We conducted a qualitative study, using a semi‐structured topic guide to interview primary care clinicians. The framework method was used for analysis, with a matrix output providing structure and enabling the authors to manage data by case and code [23]. The framework method is not aligned to any specific epistemology or ontology, allowing it to reflect the critical realist position of the research team, where multiple experiences and perceptions of a single reality are present. The consolidated criteria for reporting qualitative research (COREQ) guided the writing of this report [24] (Additional file 1 in Supporting Information S2). Ethical approval was granted by the Hull York Medical School Ethics Committee.

### 2.2 Identification and recruitment of participants

This study follows a process mapping study [25, 26], where 12 vascular surgery units were identified according to size, geographical location and participation in a quality improvement collaborative. Staff were interviewed in order to define processes in place for referrals for patients with CLTI.

Primary care clinicians who refer (or who would potentially refer) to the units where processes had been previously mapped were purposively sampled and supplemented with snowball sampling techniques. Vascular clinicians were asked to identify primary care clinicians from their personal or professional networks, and community services were emailed directly to see if any employees may be interested in participating in the study. Primary care clinicians were sampled in order to include a wide geographical spread, reflecting practice across different referral processes and different staff groups. The inclusion of nurses, podiatrists and general practitioners reflects the varied potential presentations of CLTI, which do not limit themselves to one staff group. The chosen number of 20 participants was informed by Guest et al.’s recommendations for qualitative interviews following an experiment in data saturation [27] but increased from their recommended 12 interviews to reflect a slightly higher degree of heterogeneity within our participant group.

Inclusion criteria were that the clinician had experience of working in primary care in the catchment area of a relevant vascular surgery unit. There were no exclusion criteria. No remuneration was offered for taking part in the interviews.

### 2.3 Information and consent

Potential participants were invited to take part in the qualitative interview study over email, with an explanation of the project and a Participant Information Sheet (PIS) attached. Consent was confirmed verbally both before and after the online interview, and a signed consent form was received from each participant.

### 2.4 Interviews

Interviews were carried out by EA, a female vascular surgery trainee leading the research project. She has experience in qualitative interviewing, and her clinical background involves similar techniques of information gathering and rapport development.

Interviews took place online using Microsoft Teams. Video and audio content was recorded. A topic guide (Additional file 2 in Supporting Information S3) was used, containing open questions based on the TDF, designed to elicit general and specific beliefs about the relevance of each domain to timely referral of suspected CLTI. A multidisciplinary team contributed to its design, including clinicians with expertize in vascular surgery and researchers with expertize in behaviour change and implementation science. The topic guide was subject to minor iterative alterations as the interviews progressed. Prompts were used, such as ‘tell me more’, when further explanation was considered useful.

EA's background as a vascular surgeon means she has pre‐existing assumptions around the behaviour of primary care clinicians. Using the TDF as a basis for the study helped ensure subjectivity was limited when planning and carrying out interviews and analysis [14]. Care was taken to remain neutral during the interviews and not to express opinions. A reflexive diary was used throughout, including reflective debriefing after each interview, in order to recognize and challenge assumptions.

### 2.5 Analysis

Recorded interviews were transcribed verbatim and anonymized. Directed content analysis was performed according to the framework method [23, 28]. Following familiarisation with the data, the TDF domains were used to generate a framework in Microsoft Excel, into which content from the transcribed interviews was coded by one author (EA), using a coding strategy based deductively on the TDF (Additional file 3 in Supporting Information S4), edited inductively as coding progressed. A second author (PB) independently carried out coding of a random subset representing 15% of transcripts during this process to ensure reliability of the coding strategy. PB coded utterances previously coded by EA, blinded to previous allocation and other utterances considered relevant. Responses that were coded in different domains by the researchers were discussed, and the coding strategy altered accordingly. The authors of this study were guided by Atkins et al. and their recommendations for use of the TDF [29].

One author (EA) generated belief statements using coded responses, representing the core thought of the participant. These beliefs provided detail about the role the domain is perceived to have in influencing the behaviour [15, 30]. Similar responses from different participants were coded as the same belief. This strategy was reviewed by two further authors (PB and IK) to ensure belief statements were an accurate representation of content.

Previously, relevance criteria have been used to determine which domains could be targets for future intervention [15, 18, 30]. Similar criteria were applied in this study: frequency of coding of beliefs within a domain; content of the responses of the participants coded to a particular domain (e.g., perceived as relevant or not); and conflicting beliefs coded to a domain. Relevance of domains was confirmed through discussion by the research team, considering these criteria concurrently.

## 3 RESULTS

Thirty primary care clinicians were invited to take part. Two replied to generic email invitations to community podiatry services, and one by snowball sampling via a participant who identified a colleague as someone interested in participating. The remainder were identified by vascular surgery clinicians. Twenty interviews took place. Reasons for nonparticipation included a self‐perception of unsuitability for the project, inability to find a mutually convenient time for interview within the project timeline and lack of reply to an initial approach.

Eight podiatrists, seven GPs (general practitioners) and five nurses were interviewed between November 2022 and February 2023. They referred to 11 of the 12 vascular units involved in the previous process mapping project. Interviews lasted between 30 and 56 min (mean 44 min). A total of 1450 utterances from the 20 interviews were coded into the 14 domains of the TDF. There was substantial agreement between coders, with Cohen's kappa being calculated as 0.678, indicating acceptable inter‐rater reliability [29, 31].

### 3.1 Domains reported not relevant

Five TDF domains appeared less relevant in terms of influencing recognition and referral behaviours (Supplemental Table S1). *Optimism* was not reported as an issue for referral, with faith in the local vascular team consistently described. The majority of primary care clinicians understood that not referring CLTI led to poor outcomes including amputation and death, so *beliefs about consequences* were not a barrier to referral. The conscious decision, or *intention*, to refer was driven by a perceived duty of care for most participants. *Goals* of referral were primarily relevant to improving the patient's quality of life and universal throughout the cohort. Finally, the majority of participants described using both discussion with vascular clinicians and local colleagues to inform recognition and referral decisions, indicating a lack of *social influences*, is not a barrier to referral.

### 3.2 Domains reported relevant to referral behaviour

Nine TDF domains were considered relevant to recognition and referral behaviours (Supplemental Table S2). Individual participants are referred to by a letter indicating their role and an identifying number (P# = podiatrist, N# = nurse and D# = GP).

### 3.3 Knowledge

Most participants believed they knew what CLTI was, understood the urgency and were aware of the appropriate referral pathway, but some contradicted this professed knowledge with their responses, and others stated they were not sure what CLTI was. Little teaching on the subject during clinical training was given as a reason for a lack of knowledge.

P8: So it's peripheral arterial disease, along with rest pain or an ulceration or gangrene or something like that.

P4: Yes. So we can now refer directly to vascular. We don't have to go via the GP, which is really, really brilliant, it speeds things up a bit.

N5: So obviously if it were, if I were really worried, if it were quite critical, I'd just send them to A&E [accident and emergency] and I'd ring the vascular team to say I've sent this patient to A&E. [This is not consistent with referral processes at N5’s local unit]

D7: Yeah, I have to admit that was one I had to Google, because I was… I mean, I think we all know the signs of the acute ischaemia, and that's drilled into you with your Ps and your learning in medical school. And then you've kind of got your, ohh a bit of claudication type of thing. But I think that in between that chronic limb ischemia, I wouldn't have recognised that as a descriptor and had to look it up.

D3: I had no other formal training through my foundation years or through GP training, actually. I don't think we did any specific vascular training in, through those three years of GP training.

Whilst some participants were aware of and used guidance regularly to influence their referral decisions, others were not aware of guidance or felt it was only relevant to less experienced members of staff. Over half the participants indicated local guidance or pathways would be helpful to their decision‐making process.

P9: What we tend to use, we’ve just implemented very recently, is the WIfI, so the wound, ischaemia, foot infection tool and we use the ESVS [European Society of Vascular Surgery] calculator on their app. So that does help us to guide, you know, with the referrals and things.

EA: Do you know of any guidance relating to CLTI? D6: I don't. To be totally honest, I don't. P6: I think, because I work in it a lot, I guess it's always there in my mind, I don't… But for junior staff, I think it is helpful because it's actually like a, ohh, right, OK, what am I doing and you’re following the arrows.

D4: So I think that would be really helpful, just to make sure there's clear guidance and it's really clear for everybody in… If it's not clear for me, and I still don't think it is, then I think it mustn't be clear for an awful lot of GPs in the region.

### 3.4 Environmental context and resource

All participants found recognizing and referring suspected CLTI to vascular surgery time‐consuming, describing pressure on people in the community and lack of time available to make a proper assessment of a patient.

P1: But yeah, it is. It's obviously time consuming. That's the thing.

N3: And I think because there's a lot of junior staff and new starters who have recently come in to sort of, in this job environment and working in the community, it's a lot to learn. And I think there's just so much pressure everywhere that people struggle sometimes. They panic, and they don't know which way to go.

D1: And it's like I hardly ever feel for pulses or look at feet. And that's partly again time. You know, can you take your socks and shoes off? You just lost 3 minutes.

Technology was an issue, with good technology improving the ease of referral, but poor technology such as the lack of shared notes or unreliable internet access acting as a barrier.

P4: Because we have, like, we have smartphones to take photos with. We've got laptops which we could take into patient's houses. And, you know, we could do the referral right there and then.

P7: It varies as well, in terms of if their GPs are on the same System One system as us and if the sharing's available. So sometimes I can see everything, and I can get a lot of information and I'll sort of get a better idea about, you know, might what might be going on. Sometimes I'm quite blind.

Most participants noted that patients with diabetes often have access to different pathways than those without diabetes, promoting inequality.

N2: Yes, in that, well, it's easier just to shove referrals through to podiatry, because you can just say, look, they’re diabetic and I have concerns.

Conflicting beliefs were seen regarding referral forms, with some participants finding them helpful, and others reporting downsides.

D2: Whereas other specialties, they do have proformas for different conditions. So it's like tickbox, tickbox – quite quick and easy for us to fill in, and also quick and easy for the secretaries to just send off.

D1: So I’m a member of the LMC [local medical committee], and so we often talk about these forms because the difficulty with the forms is if they're not perfectly completed, you can get rejections. And I just think that's completely, totally not helpful, you know?

### 3.5 Beliefs about capabilities

More participants described themselves and colleagues as being confident in recognition of CLTI than its referral. One reason for not being confident in referral was being perceived as not being allowed to refer to vascular surgery. The presence of written pathways and having the result of an objective measure of perfusion were highlighted as reasons for confidence.

P5: I would say I'm quite confident because I can recognise the signs.

D7: Actually, I'd probably feel quite insecure about them, because I don't think we tend to see an awful lot. There's not a lot of exposure for us, and so, you know, in terms of our pattern recognition, common things being common, common things you feel much more secure about.

N4: Yeah, I haven’t thought of doing that [referring to vascular surgery]. I don't think that’s ever kind of been said before. But no, if that was, if that were, if we knew we could do that…

N1: I guess it would feel ‐ you'd feel more confident if you were following the pathway, and going rather than just like ringing someone up and just be like, hiii.

P7: It's been really helpful since we started doing toe pressures, cause I feel like that does give me a little bit more of a potentially objective, you know, idea about what's going on.

### 3.6 Professional role and identity

Most participants stated it was their role to recognize and refer patients with suspected CLTI to vascular surgery but not their role to make decisions to *not* refer someone for assessment. GPs were often guided by nurses to refer a patient, when a nurse had suspected CLTI, but felt it was not part of their role to make the referral. Participants who felt it was not their role to refer to vascular surgery would want to be able to make referrals in future.

N5: If we're looking after them and we find it, then yeah, definitely. It's anybody's role, really if they're concerned.

EA: Are you happy to make that decision that they're not suitable for referral, or would that be something that you would look for the vascular surgery advice on? P6: Absolutely. And the GP involved, and the family. No, I certainly would not ever make that decision. I don't think it's my role.

N2: So literally I write extensively in the notes all my history taking and my concerns, and then I electronically task one of the GPs who will do the referral for me. And they tend not to ask to see the patient again. They tend to rely on what I've said and they're more than happy.

P8: I do think it would be a good thing for us to be able to do in the future, I think. We don't have that much exposure to sort of, the referrals and everything because I've never done a referral to the vascular team.

### 3.7 Skills

The main difficulty described by participants was obtaining consent from patients for referral. Vascular consultations were seen as challenging. Most were comfortable with examining patients, including objective measures, but a need was described for improvement in skills, especially in carrying out toe pressures and ABPIs.

D5: It wouldn’t prevent me referring, but it would prevent the patient accepting referral which is part of the consent process. So there are undoubtedly patients that will not go to hospital now, that we end up doing end of life stuff with at home.

D4: So, you know, I think they're the ones I think that are really difficult to then identify at what point are they actually into critical. And when actually, and what's arterial and what's actually part and parcel of their other comorbidities, and how do we get that in and communicate that appropriately before they end up being acute admissions.

N1: Yeah, so, well, if we were thinking like, we’re worried about kind of arterial problems, it would be the look of the wound, if it was located sort of foot, ankle, if it was round, defined edges, progressing fast and like raised edges. […] That's the other thing, obviously I’d do Dopplers and things, I forgot to say about that. Yeah, pulses.

P9: There's not been enough training, perhaps definitely with the lack historically of toe pressures and things like that, it's very easy to see the patient, from a podiatry perspective, put dressings on and review the patient a week later without getting to the actual cause of what, you know, how, recognizing CLTI.

### 3.8 Memory, attention and decision processes

Most participants saw the patient holistically and used their findings on history and examination as well as the wishes of the patient to help their referral decisions. Some participants suggested referral decisions should not only be based on diagnostic tools, scores or readings.

N2: We had a situation at our GP practice where an automated ABPI was done on the patient. It was done perfectly well. The ABPI was normal, but the history that the patient actually gave was not good at all. That patient should have been referred into vascular and wasn't, and ended up losing a limb.

Whether to refer a patient or not was often reported as a difficult decision, especially where patients were frail.

P2: I think what's different in podiatry now is, not necessarily just in podiatry, maybe, we see a lot more patients who perhaps there isn't anything that can be done. And they, you know, they aren't suitable for surgery. And those are the ones where I think we as clinicians probably struggle a little bit more.

### 3.9 Emotion

Despite recognition and referral leading to personal satisfaction or relief for some participants, a feeling of apprehension was described when it came to contacting the vascular team. Frustration with the process was described for many reasons, including delay in recognition, gaining consent, lack of time and feeling not listened to.

N4: Just so that we can get, I mean nothing's more satisfying than getting an ulcer healed. But also knowing that I'm doing my job and giving our patients the best treatment.

P5: Sometimes it's a relief that we've got them in, or they've agreed to go in.

D3: I think I've always had that, I think a lot of people have that nervousness about speaking to a specialist on the phone. I think it goes back to like hospital days as a junior.

P1: Well sometimes it's very frustrating, because the patient’s been like this a long time and it's never been addressed or picked up on or recognised. That's frustrating, because you always think, oh, this could have, this has been going on six months, you know.

### 3.10 Reinforcement

Previous experience with vascular surgery referrals reinforced how participants behaved. Some had had negative experiences with vascular referrals, whilst others felt supported.

D7: And we do tend to find that sometimes we get sarcastic replies back, or what's perceived as a sarcastic reply back for referrals, which then makes you again feel more insecure in what you're assessing.

P2: Our vascular surgeon is, she is really approachable. And you don't feel like that at all. And she's really, she respects what you say.

### 3.11 Behavioural regulation

Where feedback from referrals was not immediately available, participants sought the results of previous referrals in order to monitor their practice. Sometimes this was a convoluted process but participants found it helpful. Others found a clear referral pathway helped regulate referral behaviour.

N3: We have to go searching. And so I'd often look through their letters and see who they're under. And I'll just e‐mail the consultant’s secretary, or ring the secretary, or the specialist nurses. I'll ring whoever I can get hold of!

P4: So we've got our own PAD pathway that we use and that's built into our template that we use in clinics for record keeping. So there's a lot of guidance on there for staff to, you know, refer to, to make sure they're making their appropriate referrals.

## 4 DISCUSSION

This study applied the TDF to explore self‐reported influences on recognition and referral behaviour in primary care with regards to suspected CLTI. The most frequently mentioned, relevant or conflicting beliefs acting as barriers to referral behaviour adhering to published guidelines were categorised into Knowledge, Environmental context and resources, Beliefs about capabilities, Skills, Memory, Decision and attention processes, Emotions, Reinforcement and Behavioural regulation domains. Interventions designed to reduce delays in referral from primary care to vascular surgery units could include behaviour change techniques targeting these domains (Table 1) [32, 33].

**TABLE 1:** Behaviour change techniques suggested according to the TDF domain [33].

Domain	Example behaviour change technique
Knowledge (know)	Information regarding behaviour and outcome
Environmental context and resources (Env)	Environmental changes
Memory, attention and decision processes (Mem)	Self‐monitoring
	Planning and implementation
	Prompts, triggers and cues
Beliefs about capabilities (Bel Cap)	Feedback
	Increasing skills: Problem‐solving, decision‐making and goal setting
	Rehearsal of relevant skills
Professional role and identity (Id)	Social processes of encouragement, pressure and support
Skills (skill)	Graded task and starting with easy tasks
	Modelling/demonstration of behaviour by others
	Rehearsal of relevant skills
Emotion (Em)	Coping skills
	Stress management

*Note*: NB: Reinforcement and behavioural regulation domains were not used as constructs in the referenced study.

Domains of the TDF identified as irrelevant may describe enablers of recognition and referral of suspected CLTI. Future interventions should take this into account and ensure evaluation of any such intervention considers these domains alongside domains identified as relevant.

Participants' responses centred around two key issues. Firstly, participants' confidence, both in themselves and in vascular surgery, was a factor influencing recognition and referral across multiple domains. Whilst all vascular units indicated in the previous process mapping exercise that referrals would be accepted from any member of primary care staff, this was not the experience reported by participants. Some responses indicated a lack of confidence in knowledge or skills with regards to recognizing CLTI and expressed desire for written pathways to support their involvement in the referral process. Confidence to make a referral to vascular surgery was also lacking, with some participants describing tension, apprehension and previous negative experiences.

Secondly, the context in which primary care clinicians are working is extremely challenging. There are multiple demands on clinicians' time and attention, which can affect clinical behaviours, including promoting less thorough patient assessment. Poor technology can affect the ease of making referrals or seeking the results of previous referrals and further add to pressure on clinicians. Participants also noticed increasing patient complexity, including both frailty and unwillingness to consent to referral, adding challenges to their decision‐making. These perceptions are not only recognized by vascular surgery clinicians [8] but also supported by evidence from the King's Fund, who report a substantially increased workload in primary care, without being matched by increased funding or workforce, as well as increasingly complex patient care needs [34].

Our results echo the findings of previous studies in primary care, which have indicated a lack of awareness of guidelines and unclear pathways affect referral behaviour [7, 35]. Beliefs coded to the TDF domains of Knowledge, Memory and Attention and decision processes add essential detail to the findings in the literature, including the importance of easy availability of guidelines, such as those accessible within IT systems. The assurance offered to primary care clinicians by the implementation of a local pathway is also clear in our data.

Patient factors have also previously been implicated in recognition and referral of CLTI, including a delay in presentation in PAD [7], perceived poor motivation to undergo pulmonary rehabilitation in COPD (chronic obstructive pulmonary disease) [16, 17] and lack of adherence to guidelines in primary care [36]. The results of our study indicated that clinicians found the consent process challenging, and some patients would refuse referral despite explanations of the possible consequences. This has not previously been described and adds to current understanding of patient factors affecting referral behaviour.

Previous studies have used the TDF to identify other useful theories specific to the relevant domains, in order to overcome the TDF not specifying relationships between the domains [15]. In our study, the reported importance of the Knowledge and Environmental context and resource domains may be further explored with the knowledge–attitude–behaviour model [37] and the Consolidated Framework for Implementation Research (CFIR) [38], respectively.

### 4.1 Strengths and limitations

Using the TDF has allowed us to systematically identify barriers and enablers of timely recognition and referral to vascular surgery for suspected CLTI in primary care. Interviews were carried out with a diverse range of primary care clinicians, both in terms of the role and geography. Barriers and enablers reported can guide further theory‐driven research, including design, implementation and evaluation of interventions, as the TDF allows their mapping to both theory and behaviour change techniques [15, 32, 33]. Finally, the use of the TDF as a basis for the interview topic guide may have prompted the identification of barriers and facilitators of recognition and referral that participants may not have reported in an interview uninformed by a theoretical framework.

Our interview study allowed primary care clinicians to explain their own behaviour with regards to recognition and referral for suspected CLTI, but the TDF does not provide evidence of actual influences on clinical practice, and clinicians' interview data may be subject to post hoc rationalization and concern as to how they may appear to the interviewer. Quantitative work involving behaviour change interventions can provide this evidence, and the authors recommend future work in this area to explore what factors are relevant in changing practice. Our results also demonstrate the importance of patient factors in the referral process, and we have not interviewed any patients as part of this work. Doing so may have identified further barriers and facilitators of referral. We have also not investigated differences between staff groups interviewed in this study.

## 5 CONCLUSION

To our knowledge, this is the first study which has used a theoretical framework to identify barriers and enablers reported by primary care clinicians as relevant to the timely recognition and referral of patients in the community with suspected CLTI. Potential explanations are offered for known delays in the symptom to the assessment pathway. Our findings can be used to develop, implement and evaluate targeted, theory‐driven interventions to optimize the recognition and referral process mapped directly from the TDF domains.

## AUTHOR CONTRIBUTIONS

**Eleanor Atkins**: Conceptualization; data curation; formal analysis; methodology; writing – original draft; writing – review and editing. **Panagiota Birmpili**: Conceptualization; data curation; formal analysis; writing – review and editing. **Ian Kellar**: Conceptualization; formal analysis; methodology; supervision; validation; writing – review and editing. **Amundeep Johal**: Writing – review and editing. **Qiuju Li**: Writing – review and editing. **Sam Waton**: Writing – review and editing. **Jonathan Boyle**: Conceptualization; supervision; writing – review and editing. **Arun Pherwani**: Conceptualization; supervision, writing – review and editing. **Ian Chetter**: Conceptualization; supervision; writing – review and editing. **David Cromwell**: Conceptualization; supervision; writing – review and editing.

## CONFLICT OF INTEREST STATEMENT

The authors declare that they do not have any competing interests.

## ETHICS STATEMENT

Hull York Medical School Ethics Committee (ref. 21/22 32). All participants provided written consent.

## CONSENT FOR PUBLICATION

All data included in the manuscript is anonymized. Participants provided written consent for the use of their anonymized quotes in publications.

## Supporting information

Supporting Information S1

Supporting Information S2

Supporting Information S3

Supporting Information S4

## Data Availability

The data sets generated and/or analysed during the current study are not publicly available as participants were not consented for their data to be shared in this manner. Data are available from the corresponding author on reasonable request.
