# Supplementary material for: Understanding delays in chronic limb‐threatening ischaemia care: Application of the theoretical domains framework to identify factors affecting primary care clinicians' referral behaviours
Source: J Foot Ankle Res. 2024 May 4;17(2):e12015. doi: 10.1002/jfa2.12015 (PMC11296715; doi:10.1002/jfa2.12015)
Supplement: Supplementary file 1 — Supporting Information S1 [file JFA2-17-e12015-s003.docx]

**Supplementary Table 1**: Belief statements and sample quotes from non-relevant domains

| **Domain** | **Belief** | **Quote** | **Frequency (out of 20)** |
| --- | --- | --- | --- |
| Optimism (Opt) | I have faith in the vascular team to get the best outcome possible | P9: But when we get the patients into vascular, we're much more confident that, because it's a specialist service that the patients are going to get what they need | 19 |
|  |  | N4: I think just knowing that they are a good service and they will get picked up. Yeah, it's good to have that confidence in a in a service to be honest. |  |
|  |  | D6: Actually, they've been brilliant. They've been very good and I guess that faith comes from perhaps, outpatient letters about problems for patients I haven't really been involved in referring in. |  |
| Beliefs about consequences (Bel Con) | Not referring leads to bad outcomes for the foot (amputation) | P6: I, you know, you would looking at all kinds of end stage nasty wounds, looking at minor and major amputations, death, as well, if they didn't present quick enough, or be referred efficiently enough. | 18 |
|  |  | N2: Ohh well, they're gonna lose their limbs, aren't they? And I think not enough people understand that. And I'll certainly, you know, if not a whole limb, part of a limb. |  |
|  |  | D7: Equally, they might also progress to acute ischaemia and amputation. So the knock on effects are massive. |  |
|  | Not referring leads to systemic deterioration (sepsis, heart attack, death) | P9: The patient might get sepsis if they've got tissue loss, often infection with peripheral arterial disease might present differently. So it, although it's infected and people don't, might not recognise that, it might lead to sepsis, premature death. | 12 |
|  |  | N4: Yeah, I mean, I guess there could be like, well, could be death, sepsis and limb loss. Stroke, heart attack it's, yeah, not even worth thinking about |  |
|  |  | D6: And I suppose, thinking it through, that probably if you've got atherosclerosis of those arteries, you're probably gonna have them elsewhere, so renal disease, cardiac disease, cerebrovascular and the rest. |  |
| Intentions (Int) | It is my duty of care to refer if necessary | P1: I feel fine with it because it's the right thing to do | 15 |
|  |  | N1: You know, you're kind of at the moment doing everything you can, you should be doing to help them. |  |
|  |  | D2: But if you know it's gonna be for the best of the patient, then you do it. So there's no like incentive other than that, really. |  |
| Goals (Goals) | I want the patient to be revascularised | P2: We're hoping for revascularisation, so hoping that they will undertake an assessment and find out where the blockage is, and then hopefully, yeah, hopefully surgery if possible and revascularise them. | 15 |
|  |  | N3: That they'd get the interventions provided by vascular to, you know, increase the arterial supply, to get the blood flow, and save the limb, and prevent the death. |  |
|  |  | D1: And so it's just about definitive surgery, really, to kind of revascularize that limb. And then he can heal and get better, and that sort of stuff. |  |
|  | I want the patient's symptoms to improve | P4: Well, for some of them, that they're gonna end up having a revascularisation procedure. So then hopefully, you know, either the wound that they've got will heal or they'll be much more comfortable not having the rest pain or the claudication. | 15 |
|  |  | N4: Just so that we can get, I mean nothing's more satisfying than getting an ulcer healed. |  |
|  |  | D4: And secondly it's for symptom control, so someone in such severe pain that, particularly that we're struggling to really manage, then often that's a really good motivating factor to get someone referred in. |  |
|  | I want to prevent amputation | P8: Well, it might save that save their foot, save their leg, that's the end goal, really. | 12 |
|  |  | N2: Healed patients! And patients who haven't lost their limbs at the end of the day. |  |
|  |  | D3: So if I think the, in particular, if I think the limb is at risk. |  |
| Social influences (Soc) | Discussion with local colleagues informs my / their decisions | P5: I mean, the other thing is that you may get a colleague in and say, what do you think? Take a photograph, take it along and show a colleague, show a consultant. Go, I’m not happy with how this looks, what do you think? | 19 |
|  |  | N4: And yeah, like maybe like with tissue viability or even with like colleagues within the team, just like, you know what, I'm not sure about this, what do you reckon? Yeah, just to get - it's always good to get a second opinion. |  |
|  |  | D2: Like nurses are very good with, you know, like I say, they do dressings all the time, and, you know, managing all of that. And so you might ask them and see if they've, you know, what they think. You might ask your fellow GP next door what they think. |  |
|  | There is opportunity to discuss with vascular prior to referral | P5: And even sending a photograph over to the vascular team and just say, will you have a look, what do you think? You know, and they will, they're very good like that, you know | 18 |
|  |  | N5: And like I say, if I did have a little bit of uncertainty, I've always got the vascular team to give a ring and just say this is what I'm finding, what would you suggest? So there's always that back up, really, there. |  |
|  |  | D5: But, you know, if I'm unsure, I’d just pick up the phone and spoken to the consultant on call or spoken to [vascular consultant], and they tend to say we'll see them outpatients tomorrow or something along that lines. |  |

P# = podiatrist, N# = nurse, D# = GP

**Supplementary Table 2**: Belief statements and frequencies from relevant domains

| Belief | Podiatrists | Nurses | GPs | Frequency (out of 20) |
| --- | --- | --- | --- | --- |
| Knowledge (Know) |  |  |  |  |
| CLTI is an urgent condition | 6 | 4 | 4 | 14 |
| I can take an appropriate history | 7 | 3 | 4 | 14 |
| I use guidance / tools routinely (local guidance, NICE, NWCSP, WIFI, SINBAD, NEWS) | 7 | 3 | 3 | 13 |
| Local guidance / pathways would be helpful | 4 | 3 | 5 | 12 |
| I know the appropriate referral pathway(s) | 7 | 1 | 4 | 12 |
| I believe I understand what CLTI is | 7 | 3 | 1 | 11 |
| I am aware of guidance / tools but I don't use them routinely (local, NICE, NWCSP, SINBAD, WIFI) | 5 | 3 | 1 | 9 |
| I know the appropriate referral pathway(s) (incongruous belief) | 1 | 4 | 3 | 8 |
| A lack of knowledge can limit (appropriate / timely) referrals | 3 | 2 | 2 | 7 |
| GP knowledge is supported by systems |  |  | 5 | 5 |
| There is limited vascular teaching in primary care training |  |  | 5 | 5 |
| Guidance is more helpful for less experienced members of staff | 2 | 3 |  | 5 |
| I am not aware of specific guidance / tools |  |  | 4 | 4 |
| I am not confident with what CLTI is |  | 1 | 3 | 4 |
| The appearance of the limb can signify CLTI (not referring to ulcers) | 1 | 2 |  | 3 |
| I believe I understand what CLTI is (incongruous belief) | 1 | 1 | 2 | 4 |
| I am confused between acute and chronic ischaemia |  |  | 1 | 1 |
| I picked up my knowledge through experience | 1 |  |  | 1 |
| I can take an appropriate history (incongruous belief) |  | 1 |  | 1 |
|  |  |  |  |  |
| Environmental context and resources (Env) |  |  |  |  |
| Making a referral / getting advice is time-consuming | 8 | 5 | 7 | 20 |
| Diabetics have different (usually better) pathways | 7 | 4 | 6 | 17 |
| Any potential costs are irrelevant | 4 | 2 | 5 | 11 |
| Referral forms are helpful where they exist | 3 | 3 | 5 | 11 |
| Good tech facilitates referrals | 1 | 3 | 5 | 9 |
| There is a lot of pressure on people in the community |  | 1 | 7 | 8 |
| Doing a proper assessment takes time which is not always available | 2 | 2 | 3 | 7 |
| Telephone consultations are challenging |  |  | 6 | 6 |
| Shared notes are helpful where available | 3 | 1 |  | 4 |
| Standardised tools are not available for all | 3 |  | 1 | 4 |
| There is lack of time / resource for training | 1 | 1 | 2 | 4 |
| Poor connectivity (wifi / phone) hinders our capability | 3 |  |  | 3 |
| There is lack of resource for equipment | 1 | 1 | 1 | 3 |
| There is limited access to objective measures |  |  | 3 | 3 |
| Network configuration causes issues |  |  | 2 | 2 |
| Printing is difficult | 1 | 1 |  | 2 |
| A lack of equipment hinders our capability (ABPI / TP) | 1 |  |  | 1 |
| Assessment needs to be incentivised |  |  | 1 | 1 |
| Covid has delayed presentations |  |  | 1 | 1 |
| Not everyone can use required technology | 1 |  |  | 1 |
| Referral forms have downsides |  |  | 1 | 1 |
| There are many different channels of communication |  |  | 1 | 1 |
| There is pressure on vascular services | 1 |  |  | 1 |
| We are encouraged not to refer by the media |  |  | 1 | 1 |
|  |  |  |  |  |
| Beliefs about capabilities (Bel Cap) |  |  |  |  |
| I am confident in recognising CLTI | 8 | 2 | 2 | 12 |
| I am confident in referring CLTI | 7 | 3 | 1 | 11 |
| Experience gives me confidence in recognition | 6 | 2 | 1 | 9 |
| Not everyone is confident in referring CLTI | 2 | 3 | 4 | 9 |
| No matter how capable I am, there are some things I can't deal with and need referring | 2 | 1 | 5 | 8 |
| Not everyone is confident in recognising CLTI | 2 |  | 5 | 7 |
| Objective measures of perfusion increase my confidence | 3 | 3 | 1 | 7 |
| Experience gives me confidence in referral | 3 | 1 |  | 4 |
| I don't want to refer things that I can manage | 1 |  | 3 | 4 |
| Not everyone is confident to discuss consequences | 2 | 1 | 1 | 4 |
| Some staff groups are perceived to not be allowed to refer to vascular surgery by themselves or others | 2 | 1 | 1 | 4 |
| Training gives me confidence in recognition | 1 | 2 | 1 | 4 |
| Vascular ought to understand referrers' roles / capabilities | 1 | 1 | 2 | 4 |
| I can help people get appropriate treatment | 1 | 2 |  | 3 |
| Written pathways increase confidence to refer | 1 | 2 |  | 3 |
| I can understand when something isn't normal |  |  | 2 | 2 |
| I am less confident referring others' patients |  |  | 1 | 1 |
| I refer more because I have less experience |  |  | 1 | 1 |
| Lack of experience leads to lack of confidence (ABPI) |  |  | 1 | 1 |
| Training gives me confidence in referral |  | 1 |  | 1 |
|  |  |  |  |  |
| Professional role and identity (Id) |  |  |  |  |
| It is my role to refer patients to vascular | 7 | 2 | 5 | 14 |
| It is not my role to make decisions on palliating / not assessing / not treating | 7 | 4 | 2 | 13 |
| GPs are guided by nurses when vascular input is needed |  | 3 | 5 | 8 |
| Keeping updated requires personal effort | 4 | 3 | 1 | 8 |
| I can decide whether or not to refer someone (end of life) |  | 1 | 5 | 6 |
| It is not just my role to refer patients to vascular |  |  | 6 | 6 |
| I don't refer directly to vascular but I would like to | 2 | 3 |  | 5 |
| GPs / nurses need to refer to podiatry sooner | 3 |  |  | 3 |
| I can feel impostor syndrome when referring | 1 |  | 2 | 3 |
| Podiatry should be a gatekeeper for CLTI | 2 | 1 |  | 3 |
| It is not seen as my role to discuss consequences | 1 |  |  | 1 |
|  |  |  |  |  |
| Skills (Skill) |  |  |  |  |
| Obtaining consent for referral can be challenging | 8 | 4 | 5 | 17 |
| I can examine a patient appropriately including objective measures | 8 | 5 | 3 | 16 |
| There is a need for improvement in our skills | 3 | 3 | 2 | 8 |
| We improve our skills with training | 5 | 3 |  | 8 |
| Vascular consultations can be challenging | 2 |  | 5 | 7 |
| I picked up my skills through experience rather than training | 2 | 1 | 2 | 5 |
| I can examine a patient appropriately (not including objective measures) |  |  | 4 | 4 |
| Staff assessing feet are irregularly trained |  |  | 1 | 1 |
|  |  |  |  |  |
| Memory, attention and decision processes (Mem) |  |  |  |  |
| Wishes / affect of family / patient are taken into account | 6 | 2 | 6 | 14 |
| Findings on examination reinforce my decision | 5 | 3 | 1 | 9 |
| What the patient tells me reinforces my decision | 4 | 2 | 2 | 8 |
| It is difficult to know what the right thing to do is in frail patients | 5 |  | 2 | 7 |
| Referral decisions should not be based exclusively on tools / scores / readings | 3 | 2 | 2 | 7 |
| Decision making is unaffected by patient / family | 2 | 2 |  | 4 |
| Stress can divide attention |  |  | 4 | 4 |
| Regular reviews highlight deterioration which triggers referral | 3 |  |  | 3 |
| You can miss something in a consultation | 2 |  |  | 2 |
| I will err on the side of referral if I am worried |  |  | 1 | 1 |
| It needs to be severe in order to refer |  |  | 1 | 1 |
|  |  |  |  |  |
| Emotion (Em) |  |  |  |  |
| There can be apprehension when it comes to contacting the vascular team | 6 | 1 | 2 | 9 |
| Referral leads to personal satisfaction | 3 | 3 | 2 | 8 |
| I can be sad on behalf of the patient who needs a referral | 2 | 2 | 1 | 5 |
| I don't have time to feel anything, it's just doing the job | 1 | 1 | 3 | 5 |
| There is relief associated with referring | 3 | 2 |  | 5 |
| There is significant responsibility on the referrer which can be stressful | 3 | 2 |  | 5 |
| Not being able to fix everyone is frustrating | 2 |  | 1 | 3 |
| How you are feeling may affect capabilities | 1 |  | 1 | 2 |
| Stress makes me more likely to refer |  |  | 2 | 2 |
| The consent process can be frustrating |  |  | 2 | 2 |
| The referral process can be frustrating (feeling not listened to) |  |  | 2 | 2 |
| The referral process can be frustrating (lack of time) |  |  | 2 | 2 |
| There can be anxiety associated with decision making | 1 |  | 1 | 2 |
| An unclear pathway is demoralising |  |  | 1 | 1 |
| I feel guilty if the patient doesn't turn up | 1 |  |  | 1 |
|  |  |  |  |  |
| Reinforcement (Reinf) |  |  |  |  |
| Some referrals can get a negative reaction from vascular | 5 | 2 | 4 | 11 |
| Previous experience reinforces decisions | 5 | 1 | 3 | 9 |
| We are supported by the vascular team | 6 | 2 |  | 8 |
| There are no national incentives to diagnose / refer |  |  | 1 | 1 |
| We are trusted by the team who make referrals to vascular |  | 1 |  | 1 |
|  |  |  |  |  |
| Behavioural regulation (Beh Reg) |  |  |  |  |
| I seek results of past referrals to self-monitor | 6 | 4 |  | 10 |
| We do the same thing with every patient | 5 | 2 |  | 7 |
| A clear referral pathway / criteria really helps | 4 |  | 2 | 6 |
| Referrals are audited |  |  | 1 | 1 |
| Regular reviews highlight deterioration which triggers referral | 1 |  |  | 1 |
| Structured referral tools like SBAR help | 1 |  |  | 1 |
