# Supplementary material for: Understanding delays in chronic limb‐threatening ischaemia care: Application of the theoretical domains framework to identify factors affecting primary care clinicians' referral behaviours
Source: J Foot Ankle Res. 2024 May 4;17(2):e12015. doi: 10.1002/jfa2.12015 (PMC11296715; doi:10.1002/jfa2.12015)
Supplement: Supplementary file 2 — Supporting Information S2 [file JFA2-17-e12015-s002.docx]

| **Domain 1: research team and reflexivity** | | |
| --- | --- | --- |
| **Personal characteristics** | | |
| 1. Interviewer/facilitator | Which author(s) conducted the interview or focus group? | One researcher (EA) conducted the interviews |
| 2. Credentials | What were the researcher’s credentials? (e.g. PhD, MD) | EA is working towards a postgraduate qualification |
| 3. Occupation | What was their occupation at the time of the study? | EA is a vascular surgeon |
| 4. Gender | Was the researcher male or female? | EA is female |
| 5. Experience and training | What experience or training did the researcher have? | EA has been involved in previous qualitative research |
| **Relationship with participants** | | |
| 6. Relationship established | Was a relationship established prior to study commencement? | Some participants were known to EA prior to the study commencement |
| 7. Participant knowledge of the interviewer | What did the participants know about the researcher? (e.g. personal goals, reasons for doing the research) | Participants knew EA’s background as a vascular surgeon, and understood the purpose of the research |
| 8. Interviewer characteristics | What characteristics were reported about the interviewer/facilitator? (e.g. bias, assumptions, reasons and interests in the research topic) | As a vascular surgeon, EA had pre-existing assumptions around the study. These were regularly reflected on during the research process |
| **Domain 2: study design** | | |
| **Theoretical framework** | | |
| 9. Methodological orientation and theory | What methodological orientation was stated to underpin the study? (e.g. grounded theory, discourse analysis, ethnography, phenomenology, content analysis) | Directed content analysis was performed according to the framework method |
| **Participant selection** | | |
| 10. Sampling | How were participants selected? (e.g. purposive, convenience, consecutive, snowball) | Primary care clinicians who refer (or who would potentially refer) into the units where processes had been previously mapped were purposively sampled, supplemented with snowball sampling techniques |
| 11. Method of approach | How were participants approached? (e.g. face to face, telephone, mail, e-mail) | Potential participants were invited to take part in the qualitative interview study over email |
| 12. Sample size | How many participants were in the study? | 20 |
| 13. Non-participation | How many people refused to participate or dropped out? Reasons? | None dropped out or rescinded consent at a later stage |
| **Setting** | | |
| 14. Setting of data collection | Where was the data collected? (e.g. home, clinic, workplace) | Interviews were carried out online using Microsoft Teams |
| 15. Presence of non-participants | Was anyone else present besides the participants and researchers? | Non-participants were not present |
| 16. Description of sample | What are the important characteristics of the sample? (e.g. demographic data, date) | Eight podiatrists, seven GPs and five nurses were interviewed between November 2022 and February 2023 |
| **Data collection** | | |
| 17. Interview guide | Were questions, prompts, guides provided by the authors? Was it pilot tested? | A topic guide (Additional file 2) was used, based on the TDF, designed to elicit general and specific beliefs about the relevance of each domain to timely referral of suspected CLTI. It was not pilot tested. The topic guide was subject to minor iterative alterations as the interviews progressed. Prompts were used, such as “tell me more”, when further explanation was considered useful |
| 18. Repeat interviews | Were repeat interviews carried out? If yes, how many? | No |
| 19. Audio/visual recording | Did the research use audio or visual recording to collect the data? | Each interview was audio and video recorded |
| 20. Field notes | Were field notes made during and/or after the interview or focus group? | A reflexive diary was kept, and individual reflections written after each interview |
| 21. Duration | What was the duration of the interviews or focus group? | Interviews lasted between 30 and 56 minutes (mean 44 minutes) |
| 22. Data saturation | Was data saturation discussed? | The chosen number of 20 participants was informed by Guest et al’s recommendations for qualitative interviews following an experiment in data saturation, but increased from their recommended 12 interviews to reflect a slightly higher degree of heterogeneity within our participant group |
| 23. Transcripts returned | Were transcripts returned to participants for comment and/or correction? | No |
| **Domain 3: analysis and findings** | | |
| **Data analysis** | | |
| 24. Number of data coders | How many data coders coded the data? | Following familiarisation with the data, the TDF domains were used to generate a framework in Microsoft Excel, into which content from the transcribed interviews was coded by one author (EA), using a coding strategy developed based deductively on the TDF, and edited inductively as coding progressed. A second author (PB) independently carried out coding of 15% of transcripts during this process to ensure reliability of the coding strategy. PB coded utterances previously coded by EA, blinded to previous allocation, and other utterances thought to be relevant. |
| 25. Description of the coding tree | Did authors provide a description of the coding tree? | The coding strategy is attached as Additional File 3 |
| 26. Derivation of themes | Were themes identified in advance or derived from the data? | No themes were identified in this work |
| 27. Software | What software, if applicable, was used to manage the data? | Microsoft Excel |
| 28. Participant checking | Did participants provide feedback on the findings? | No |
| **Reporting** | | |
| 29. Quotations presented | Were participant quotations presented to illustrate the themes/findings? Was each quotation identified? (e.g. participant number) | See Results section. Each quote was identified with participant number |
| 30. Data and findings consistent | Was there consistency between the data presented and the findings? | When reviewed against the initial transcripts, the study findings accurately reflect the data collected |
| 31. Clarity of major themes | Were major themes clearly presented in the findings? | Relevant domains are presented in the Results section |
| 32. Clarity of minor themes | Is there a description of diverse cases or discussion of minor themes? | Relevant domains are presented in the Results section |
