# Supplementary material for: Understanding delays in chronic limb‐threatening ischaemia care: Application of the theoretical domains framework to identify factors affecting primary care clinicians' referral behaviours
Source: J Foot Ankle Res. 2024 May 4;17(2):e12015. doi: 10.1002/jfa2.12015 (PMC11296715; doi:10.1002/jfa2.12015)
Supplement: Supplementary file 3 — Supporting Information S3 [file JFA2-17-e12015-s004.docx]

**Indicative interview topic guide: primary care clinicians**

Questions are likely to include the following:

- Introduction, aims of interview, safety information, consent
- Gathering data on participant
  - Professional role
  - Number of vascular referrals over past 6 months? Year?

Using Theoretical Domains Framework to assess knowledge, capability and motivation

- Knowledge
  - What do you understand by the term “chronic limb threatening ischaemia (CLTI)”?
  - Do you use, or know of any guidance relating to CLTI?
  - PROMPTS: Local or national guidance on when and how to refer?
  - Tell me about any training or teaching you’ve had on vascular disease
    - PROMPTS: Any training during your qualification, since your qualification, any e-learning?
- Skills
  - Can you describe how you would take a history from a patient with foot pain or an ulcer who you suspect might have vascular problems?
  - How would you examine a patient who you suspected of having CLTI?
  - Do you carry out ABPIs or toe pressures? If not, why not?
  - If you decided the patient needed further assessment, can you tell me about how you’d refer in your practice?
    - Are there features of the patient / presentation that might change your approach?
    - PROMPTS: Does it matter if they are diabetic or not?
- Professional role and identity
  - Is it your role to refer patients with CLTI to vascular surgery?
    - If not, who should do it?
- Beliefs about capabilities
  - How confident do you feel in recognising CLTI?
    - What makes you confident / not confident?
  - How confident do you feel in referring CLTI?
    - Why?
    - Does the person you are referring to make a difference?
  - Have you had any issues with referrals for CLTI in the past?
    - Have you met who you’re referring to?
- Optimism
- How confident are you that referral will have the best possible outcome for the patient?
- How much faith do you have in the vascular team that you’re referring to?
- Beliefs about consequences
  - What would happen to the patient if the referral wasn’t made? If they declined, for example, or if they presented late?
  - What would happen if the referral process worked for patients with CLTI?
    - What would happen to the patient?
    - Would there be any other consequences?
  - Do you tell the patients about consequences?
- Reinforcement
  - What might reinforce, or strengthen your decision to make a referral?
    - PROMPT: Would you discuss with a local colleague? Would you discuss with a hospital colleague?
  - What factors might hinder this decision process, or make you less likely to refer?
  - Have you ever had feedback on a referral?
    - How did that make you feel? Why?
    - Do you think feedback would be helpful?
- Intentions
  - What do you hope to achieve with a referral for a patient with CLTI?
  - Do you anticipate any problems?
  - PROMPTS: Any patient factors that might cause a problem?
- Memory, attention and decision processes
  - What is the alternative to referral?
    - When would you choose to do the alternative? Why?
    - Are there patient factors that affect your decision?
    - PROMPTS: What would you do with a palliative, or end of life patient?
  - What do you think might help you make the decision to refer, or not?
  - PROMPTS: We talked about guidance earlier, is there anything like that? A decision aid?
  - Would you refer to anyone but the vascular surgeons? Podiatry?
- Goals
  - What motivates you to refer a patient? Why would you do this?
  - Are there any incentives for referrals? Any benefit to you or the practice you work in?
  - Are there any costs for referrals?
- Environmental context and resources
  - Are there any factors in the environment you work in that affect the referral process?
    - PROMPTS: Like how busy the clinic is, time available to call / email?
    - PROMPTS: Form of consultation (?telephone), pressure on GP in terms of decision making / capacity for risk?
  - Are there any environmental factors from the hospital end that affect the referral process?
    - PROMPTS: Do you have to fill in an online form, waiting for switch to bleep the reg etc.
- Social influences
  - Do you know what your colleagues do with patients with suspected CLTI?
  - Do you discuss referrals as a group?
  - Are you encouraged or discouraged to make referrals?
- Emotion
  - How does referring / having to refer a patient make you feel?
    - Why?
  - Do patients’ and families’ emotions affect your decision making?
  - Does how you feel at the time affect what you might do?
- Behavioural regulation
- How do you ensure that your referral behaviour is appropriate to the situation?
- Do you have personal strategies to standardise your practice?

Questions not immediately related to TDF

- Pathways
  - What would your ideal pathway for referring a patient to vascular surgery look like?
  - PROMPT: Who would you like to refer to, and how?
  - How could your current pathway be better?
- Potential resources
  - What do you think would help support you referring patients with suspected CLTI?
    - Why will this work?
  - Is there anything you think wouldn’t help? Anything that’s already been tried?
    - Why?
  - Do you know of any resources already in place in other specialties?
    - Why are they good / bad?

Close

- Anything you’d like to add, or anything that we haven’t covered?

Questioning here will be adapted in response to ongoing iterative analysis of the interview data.
