# Supplementary material for: Understanding delays in chronic limb‐threatening ischaemia care: Application of the theoretical domains framework to identify factors affecting primary care clinicians' referral behaviours
Source: J Foot Ankle Res. 2024 May 4;17(2):e12015. doi: 10.1002/jfa2.12015 (PMC11296715; doi:10.1002/jfa2.12015)
Supplement: Supplementary file 4 — Supporting Information S4 [file JFA2-17-e12015-s001.docx]

**TDF coding manual**

| **Domain Definition** | **Constructs** | **Notes** |
| --- | --- | --- |
| **Knowledge (know)**  An awareness of the existence of something | Knowledge (including knowledge of condition / scientific rationale)  Procedural knowledge  Knowledge of task environment | - Knowledge of CLTI (definition, urgency) - Knowledge of referral pathways - Knowledge of what to ask in a history - Knowledge and use of guidelines / pathways (including desire for criteria / pathway) - How knowledge is gained (teaching) - Support for knowledge - Consequences of a lack of knowledge   (Knowledge may be both correct and incorrect knowledge – eg. incorrect definition of CLTI, or admitting a lack of understanding) |
| **Skills (skills)**  An ability or proficiency acquired through practice | Skills  Skill development  Competence  Ability  Interpersonal skills  Practice  Skill assessment | - Ability to examine a patient - Consultation skills (consent for referral) - Challenges needing to be overcome during consultations - How skills have been gained (training / experience) - Need for improvement in skills |
| **Social / professional role & identity (id)**  A coherent set of behaviours & displayed personal qualities of an individual in a social or work setting | Professional identity  Professional role  Social identity  Identity  Professional boundaries  Professional confidence  Group identity  Leadership  Organisational commitment | - Clinicians’ expressions about their own professional identity / job / role / professional boundaries - Comparisons of their role with that of other professionals - How different professions work together - The role of personal effort (seeking out training) - (Not) others’ role / responsibility - Impostor syndrome |
| **Beliefs about capabilities (bel cap)**  Acceptance of the truth, reality, or validity about an ability, talent, or facility that a person can put to constructive use | Self-confidence  Perceived competence  Self-efficacy  Perceived behavioural control  Beliefs  Self-esteem  Empowerment  Professional confidence | - Perceptions about their own competence / confidence in assessing / managing CLTI - What gives them the competence / confidence to assess / manage CLTI - Thoughts on others’ competence / confidence with regards to CLTI - Opinions on what they can manage vs need for referral - Perceived competence / confidence compared to others |
| **Optimism (opt)**  The confidence that things will happen for the best or that desired goals will be attained | Optimism  Pessimism  Unrealistic optimism | - Degree of faith in the vascular team that the best possible outcome will be achieved - Perceptions of waiting times - Things that change optimism (eg. network configuration, meeting people) |
| **Beliefs about consequences (bel con)**  Acceptance of the truth, reality, or validity about outcomes of a behaviour in a given situation | Beliefs  Outcome expectancies  Anticipated regret  Consequences | - Perceptions of what happens if CLTI is / isn’t recognised / referred (negative or positive, related to patient, self or work environment) - Perceptions of the vascular surgery assessment process (negative or positive) - Beliefs relating to the treatment of CLTI and its outcomes - Beliefs relating to the referral process for CLTI |
| **Reinforcement (reinf)**  Increasing the probability of a response by arranging a dependent relationship, or contingency, between the response & a given stimulus | Rewards  Incentives  Punishment  Consequents | - Previous experience of referrals affecting how the participant thinks of the referral process (positive or negative) - Degree of trust from vascular team |
| **Intentions (int)**  A conscious decision to perform a behaviour or a resolve to act in a certain way | Stability of intentions  Stages of change | - What the decision to refer a patient comes down to (duty of care) - Decisions being made case by case – not letting external factors affect each consultation - Ensuring good quality of referral |
| **Memory, attention & decision processes (mem)**  The ability to retain information, focus selectively on aspects of the environment & choose between two or more alternatives | Memory  Attention  Decision making  Cognitive overload / tiredness | - How decisions are made on whether to refer or not (findings on examination, tools / scores, patient history) - Effect of family or patient wishes on decisions - Effect of stress - Situations in which decision making is difficult (frailty) |
| **Goals (goals)**  Mental representations of outcomes or end states that an individual wants to achieve | Goals  Implementation intention | - What participants are hoping to achieve by referring a patient (affecting self / patient) |
| **Environmental context & resources (env)**  Any circumstance of a person's situation or environment that discourages or encourages the development of skills & abilities, independence, social competence & adaptive behaviour | Environmental stressors  Resources / material resources  Organisational culture  Person x environment interaction  Barriers and facilitators | - Describing the presence or absence of resources / equipment / services / clinicians / organisational structures which facilitate / impede performing the behaviour – eg: - Equipment - Technology - Referral forms / tools - Time - Clinical demands / pressure - Shared notes - Network configuration - Means of communication between primary and secondary care - Presence of alternative pathways for some patients (diabetics) - Views on the costs associated with the referral - Effect of form of consultation on the assessment of CLTI - Effect of Covid |
| **Social influences (soc)**  Those interpersonal processes that can cause individuals to change their thoughts, feelings, or behaviours | Social pressure / norms / comparisons  Group conformity / norms  Social support  Power  Intergroup conflict  Alienation  Group identity  Modelling | - Effects of contact with the vascular team (including different staff groups) - Feedback given (or not) following referrals - What local colleagues do (same or different to participant) - Formal or informal learning taking place - Communication between primary and secondary care (the act of communication as opposed to means of) - Communication within primary care - Perceptions of discussing with different types of vascular clinician (nurse v. surgeon) |
| **Emotion (em)**  A complex reaction pattern, involving experiential, behavioural, & physiological elements, by which the individual attempts to deal with a personally significant matter or event) | Fear  Anxiety  Positive / negative affect  Stress  Depression  Burnout | Reference to any emotions experienced by the participant with reference to the assessment / referral process for CLTI:   - Anxiety - Apprehension - Frustration - Relief - Satisfaction - No feelings |
| **Behavioural regulation (beh reg)**  Anything aimed at managing or changing objectively observed or measured actions | Self-monitoring  Breaking habits  Action planning | - Statements on how the participants self-monitor - Statements about processes / prompts in place which help standardise behaviour (pathways / criteria / tools) - Statements about personal strategies to standardise behaviour (doing the same thing with each patient) - Processes in place to audit behaviour |

Coding manual based on definitions provided in Cane, O’Connor & Michie (2012). TDF, Theoretical Domains Framework
